# Experimental validation of auxetic stent designs: three-point bending of 3D printed Titanium prototypes

**DOI:** 10.3389/fmedt.2024.1388207

**Published:** 2024-05-06

**Authors:** Rahul Vellaparambil, Woo-Suck Han, Pierluigi Di Giovanni, Stéphane Avril

**Affiliations:** ^1^ Mines Saint-Etienne, Université Jean Monnet Saint-Etienne, INSERM, SAINBIOSE U1059, Saint-Etienne, France; ^2^ Research and Development Department, HSL S.R.L, Trento, Italy

**Keywords:** auxetic stent, finite element analysis, three-point bending, DMLS additive manufacturing, endovascular stent, experimental validation

## Abstract

**Introduction:**

Numerical simulations have demonstrated the superior bending flexibility of auxetic stents compared to conventional stent designs for endovascular procedures. However, conventional stent manufacturing techniques struggle to produce complex auxetic stent designs, fueling the adoption of additive manufacturing techniques.

**Methods:**

In this study, we employed DMLS additive manufacturing to create Titanium Ti64 alloy stent prototypes based on auxetic stent designs investigated in a previous study. These prototypes were then subjected to experimental three-point bending tests.

**Result:**

The experimental results were replicated using a finite element model, which showed remarkable accuracy in predicting the bending flexibility of four auxetic stents and two conventional stents.

**Discussion:**

Although this validation study demonstrates the promising potential of DMLS and other additive manufacturing methods for fabricating auxetic stents, further optimization of current stent design limitations and the incorporation of post-processing techniques are essential to enhance the reliability of these additive manufacturing processes.

## 1 Introduction

Auxetic metamaterials possess a negative Poisson's ratio (NPR), which can be exemplified by its ability to expand or contract when subjected to tension or compression respectively (1). These metamaterials provide advantages in comparison to conventional materials [possessing a positive Poisson's ratio (PPR)] such as increased shear resistance, increased indentation resistance, good energy absorption, hardness and fracture toughness (2), which are all appreciated for vascular stenting applications. Auxetic metamaterials have been proposed for stenting applications due to superior advantages over traditional vascular stent designs, such as removal of foreshortening post-stent enlargement after deployment in artery (3). Considering the NPR property of arterial endothelium (4), an auxetic vascular stent could be a better fit to the arterial tissue in terms of conformability. Self-expanding stents derived from topologically optimized auxetic unit cells demonstrate adaptive stiffness, favorable flexibility and conformability to deter stent mal-apposition and inappropriate stent expansion (5). Conventional stent manufacturing methods are not suited to fabricate vascular stents derived from auxetic unit cells due to their intricate morphology (6). Additive Manufacturing (AM) techniques are perfectly suited to fabricate personalized stents with morphological complexity, with increasing research interest shown for the fabrication of AM vascular stents in the last decade (7–9).

In recent years, there has been a surge of research publications focusing on the development of AM stents derived from auxetic unit cells for various medical applications. Wu et al. (10) utilized fused deposition modelling (FDM) technique to fabricate a stent with arrowhead auxetic unit cell design in polylactic acid (PLA) material, which enabled the stent to possess self-expanding attributes. Ruan et al. (11) developed anti-chiral-re-entrant hybrid auxetic unit based stents by stereolithography (SLA) technology with photopolymer resin. Geng et al. (4) developed novel chiral stent designs with auxetic features and generated prototypes from nylon powder based selective laser sintering (SLS) technology. Xue et al. (5) designed a topological optimized stent with chiral based auxetic unit cell, which was fabricated using a multi-material jetting AM technique. FDM technology was implemented by Lin et al. (12) to generate a shape memory PLA based stent composed of optimized concave honeycomb NPR unit cells.

Wang et al. (13) developed stents with zero Poisson's ratio (ZPR) structure and applied an innovative screw extrusion based 3D printing system to generate ZPR stents from polycaprolactone (PCL) material. Guo et al. (14) designed stents comprising of NPR convex-concave honeycomb unit cells and fabricated these stents using SLS AM technique in nylon material. Jiang et al. (15) employed polyjet AM techniques with photocurable resins to generate stents comprising of auxetic tubular lattice unit cells. Chen et al. (6) employed laser powder bed fusion (LPBF) AM technology to assemble stents comprised of anti-tetra-chiral auxetic unit cells with 316l stainless steel powder raw material. Xiao et al. (16) applied high-resolution projection micro-stereolithography AM technique to generate stents with planar anti-chiral NPR unit cells, followed with gold nano thin film coating applied in post-processing steps to enhance radial strengthening. Abbaslou et al. (17) generated 3D printed stents with novel re-entrant meta-tri-chiral auxetic unit cell design using FDM technology with PLA filament. Pramanik et al. (18) utilized LPBF AM technology to fabricate auxetic stents based on Ti2448 metal alloy.

Therefore, considering the emphasis on low strut thickness and intricate stent design, FDM, SLA and LPBF AM techniques appear to be among the most popular choices for fabricating stents derived from auxetic unit cells. Metallic stents constructed from auxetic unit cells are typically fabricated through an AM technique, a process that precisely fuses powdered metal layers on a powder bed to establish the desired stent geometry (19). This AM technique was referred to as SLS, Direct Metal Laser Sintering (DMLS) and Selective Laser Melting (SLM) for metal AM manufacturing purposes for patent purposes by different manufacturers (20). Generally in previous literature mentioned above, there is a research gap concerning the feasibility of DMLS metal AM technique to fabricate stent prototypes based on different auxetic designs considering the challenges with its intricate morphology.

In a previous work from our group (21), we explored the bending flexibility of 4 auxetic stent designs (re-entrant (RE), chiral, chiral-re-entrant hybrid (CRE) and chiral-star (CS) unit cells) and 2 conventional stent designs (diamond (DIA) and honeycomb (HCB) unit cells) with finite element (FE) modelling. We showed that three stent designs derived from auxetic unit cells (RE, CRE, CS) offer better flexibility in comparison to stents derived from traditional diamond unit cells for endovascular applications based on numerical simulations. In this study, we explore the potential of DMLS AM technology to fabricate titanium alloy prototypes based on the previously mentioned 6 stent designs and demonstrate the accuracy of our FE model in predicting their bending behavior.

## 2 Materials and methods

Six different stent designs based on specific unit cells from our earlier work, have been reconstructed with new strut thickness, stent length and stent diameter as mentioned in the Table 1. DMLS process was implemented to develop Titanium alloy prototypes based on the six individual stent designs. An inspection of the average strut thickness of each as-printed stent is conducted to assess any deviations and accordingly these deviations are implemented in their respective FE models to avoid any inaccurate prediction of their respective mechanical behavior. A three-point bending test protocol is established as per ASTM standards to capture the bending behavior of the prototypes. Finally, the three-point bending test protocol is reconstructed using a FE model and the numerical results are compared with the experimental testing results of the Titanium stent prototypes.

**Table 1:** Dimensions of stent geometries as-designed (strut thickness has been altered for individual stent designs post-examination of as-printed stents).

Stent length (mm)	Stent diameter (mm)	Strut thickness *(mm)	Mesh element type
82	16	0.4	B31

### 2.1 Stent designs

**Figure 1:**
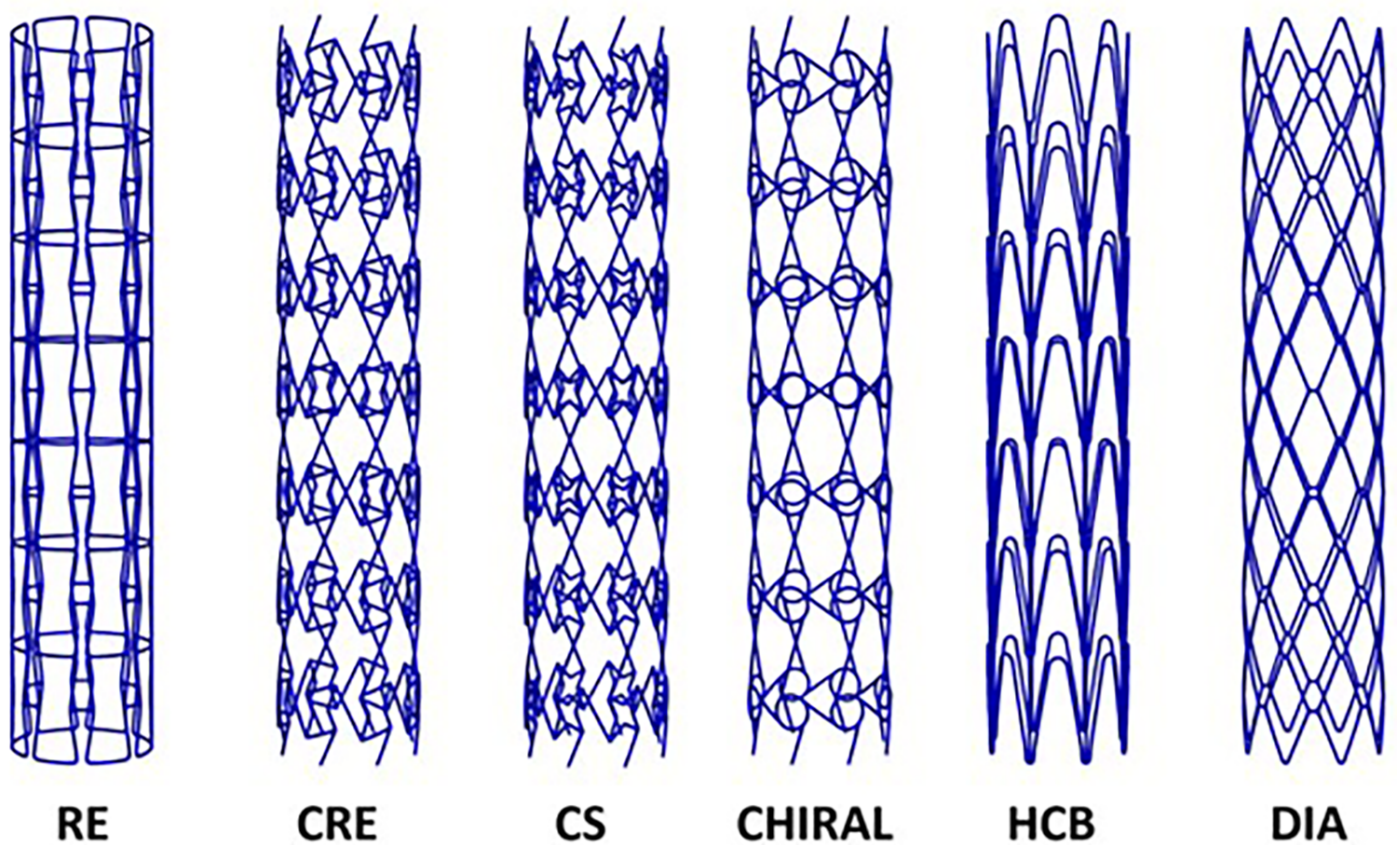
Three-dimensional models of 6 stent designs considered in this study.

All stent geometries were generated on Abaqus (v2020, Simulia, Dassault Systemes) with the wrap mesh plugin application to possess eight unit cells in the circumferential directions and seven unit cells in the longitudinal direction as observed in Figure 1. All stents were modeled using 2-node linear B31 beam elements (21). An element size of 0.1 mm was selected for all stent designs after a preliminary mesh sensitivity analysis and the corresponding number of mesh elements for each stent design has been enlisted in Table 2. Sharp edges observed on the stent geometries were modified using CATIA (Dassault Systèmes) software under feedback with Electro Optical Systems (EOS GmbH, Germany) AM engineers and exported in STL format for AM fabrication.

**Table 2:** Mesh element discretization for all stent designs considered in this study.

Stent type	Number of Mesh elements
RE	18,000
CRE	18,704
CS	19,376
CHIRAL	14,336
DIA	15,096
HCB	19,792

### 2.2 DMLS printing of titanium alloy stent prototypes

**Figure 2:**
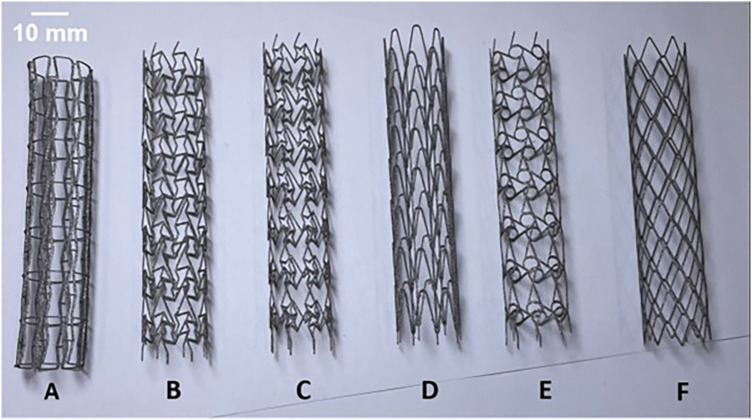
As-built DMLS printed Titanium alloy prototypes of RE (**A**), CRE (**B**), CS (**C**), HCB (**D**), CHIRAL (**E**), DIA (**F**) stent designs.

All stent prototypes observed in Figure 2, were produced using DMLS additive manufacturing technology on an industrial 3D printing EOSINT M 290 machine (EOS GmbH, Germany) with Titanium alloy (Ti-6Al-4V Grade 5) powder, EOS Titanium Ti64 (EOS Ti64 material datasheet, 22). Based on the CAD data provided to EOS engineers, build supports for file preparation were completed using Magics (Materialise, Belgium) software for each individual stent prototype alongside default manufacturing process parameters for selected material. The fabrication of prototypes were conducted in a vertical build direction with a layer thickness of 30 µm in an argon gas environment using a ytterbium laser source with a maximum laser power of 400 W. The minimum achievable wall thickness for the prototypes with DMLS was estimated to be within a range of 0.3 mm—0.4 mm and our targeted wall thickness was set at 0.4 mm as per Table 1. Following removal of the prototypes from the build-plate, the surface quality was examined using optical microscopy and the wall thickness of the stent prototypes was validated using a vernier caliper to assess the under/over-sizing of the as-built prototypes from the original as-designed stents.

### 2.3 Three-point bending test: experimental protocol

**Figure 3:**
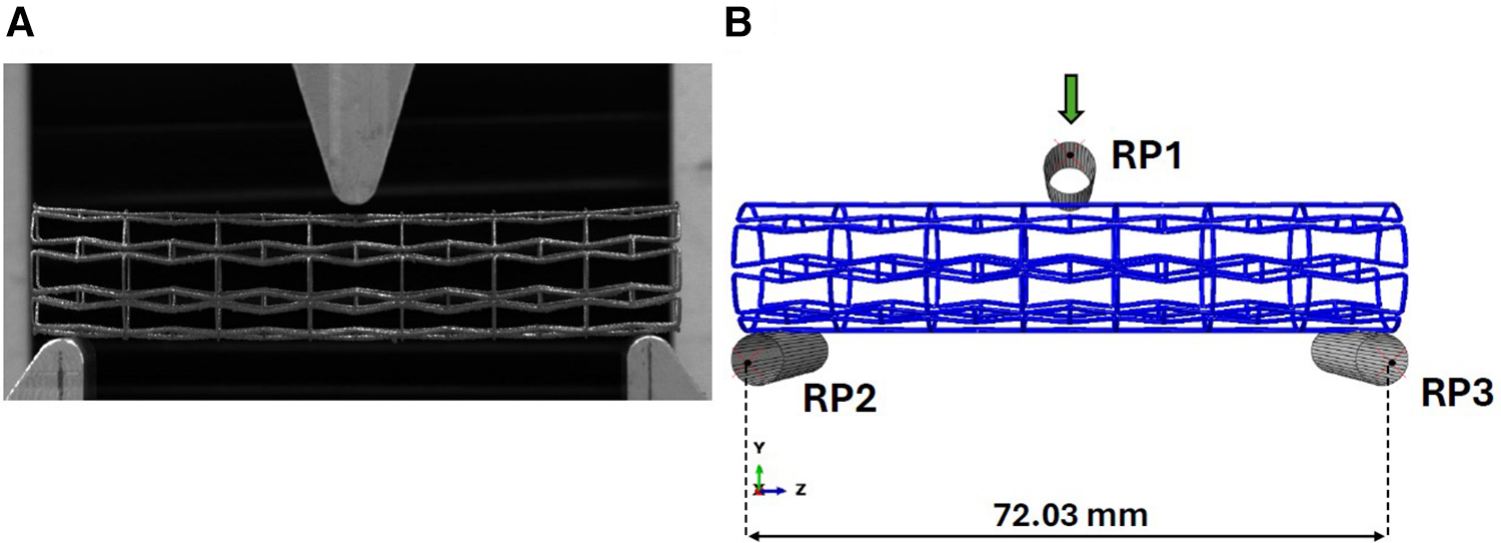
(**A**) Experimental setup for the three-point bend testing of titanium prototype. (**B**) Abaqus model for three-point bend testing of titanium prototypes.

We have replicated the testing protocol elaborated in our previous work as per ASTM standard F2606-08 2021 (23) to assess the bending flexibility of our 3D printed titanium prototypes and develop a FE model to replicate the experimental test. Compliant to ASTM F2606-08 2021 guidelines for a stent diameter of 16 mm, a span length of 73.03 mm and maximum deflection of 10 mm were established. We utilized an Instron universal tester machine (Instron, MA, USA) to apply a maximum bending deflection of 10 mm at rate of 0.1 mm/s as seen in Figure 3A, with a 500N load cell (Instron, MA, USA) attached to the system recording the load applied to the stent and displacement at a sampling rate of 50 Hz. Based on the data extracted from the testing of 3 samples per each individual stent design (23), bending load vs. deflection curves were plotted to demonstrate their respective bending flexibility performance.

### 2.4 Three-point bending test: FE model

To simulate the bending test, a FE model was developed using Abaqus Explicit solver v2020 (Simulia, Dassault Systemes, France) with identical boundary conditions enforced in our earlier work (21). The indenter and fixed loading supports in the experimental setup have been reconstructed as rigid cylinders as observed in Figure 3B. Reference points (RP1, RP2, RP3) as denoted in Figure 3B are enabled to control the cylinders using rigid body constraints, with the upper cylinder being the only object allowed to move in the vertical direction during the loading step and the bottom two cylinders being fully constrained. A contact condition was enforced between the stent and the three cylinders with a friction coefficient magnitude of 0.15 (24).

Reaction force magnitudes were recorded from RP1 reference point to generate bending force vs. deflection plots for comparison with experimental results. A mass scaling method with a target time increment of 1e-6 s was employed to maintain a quasi-static environment during each analysis. This step was taken to minimize the impact of inertial effects, which was verified by ensuring that the ratio of kinetic energy to internal energy remained below 1%. The bending stiffness (EI) of the stents can be determined utilizing equation (1), where L signifies the fixed span, F represents the bending load, and f signifies the bending deformation, as outlined by Wang et al. (24) below:

(1) $$\mathrm{EI}\;=\;\frac{F.\;L^{3}}{48f}$$

### 2.5 Material model for Titanium alloy (Ti-6Al-4V) stent prototypes

Given that the titanium alloy prototypes were produced with strut thickness at the lowest possible fabrication threshold (0.4 mm), producing similarly thin titanium alloy dog-bone samples feasible for uniaxial testing would be time-consuming and cost-prohibitive. Furthermore such samples would be unreliable due to surface distortions caused by residual stresses during fabrication. On reviewing the literature reviewing mechanical testing of DMLS or selective laser melting (SLM) printed prototypes based on titanium alloy (Ti-6Al-4V) material with a strut thickness of 0.4 mm, we found that the Young's modulus magnitude was observed to be within 107 ± 3 GPa during its elastic response (25). Based on this experimental data, we decided to implement a simple linear elastic constitutive model to represent Ti-6Al-4V with Young's modulus E = 107 GPa and Poisson's ratio *ν* = 0.31 (26) and density as 4.41 g/cm^3^ (22).

## 3 Results

### 3.1 Surface quality and thickness examination of stent prototypes

**Figure 4:**
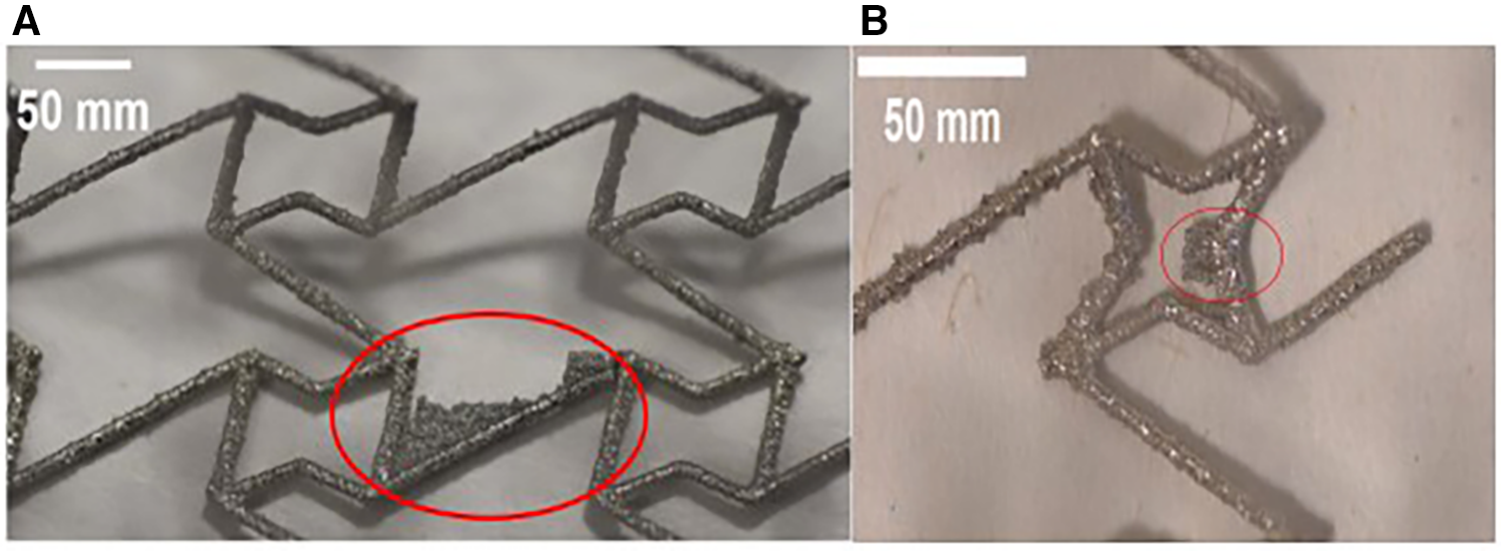
Optical microscope images of stent struts reveal residual fragments of support structures (highlighted in red) on both (**A**) CRE and (**B**) CS prototypes.

The surface quality of most prototypes was found to be satisfactory, exhibiting some loosely adhered particles due to a typical characteristic of the DMLS process. However, the CRE and CS prototypes deviated from this observation, revealing residual support structures as shown in Figure 4. Given the potential risk of stent fracture associated with these fragments, affected prototypes were not subjected to any post-processing.

To assess the deviation in wall-thickness of as-printed stent struts from the as-designed stent struts, we recorded the wall-thickness at 20 different points for 3 prototypes of each stent design that was selected for the three-point bending test. Using this data, we were able to observe the average wall thickness for each stent design which was incorporated into their respective FE model. On examining the mean thickness results noted in Table 3, RE & Chiral were oversized by 5% and CRE & DIA were undersized by 5% compared to original CAD data.

**Table 3:** Mean thickness data compiled from 3 samples per stent design.

Stent design	Wall thickness (mm)	
	Average	Standard deviation
RE	0.42	0.024
CRE	0.38	0.081
CS	0.40	0.042
HCB	0.40	0.131
DIA	0.38	0.172
CHIRAL	0.42	0.128

### 3.2 Three point bend test results: comparison between FEA and experimental testing

**Figure 5:**
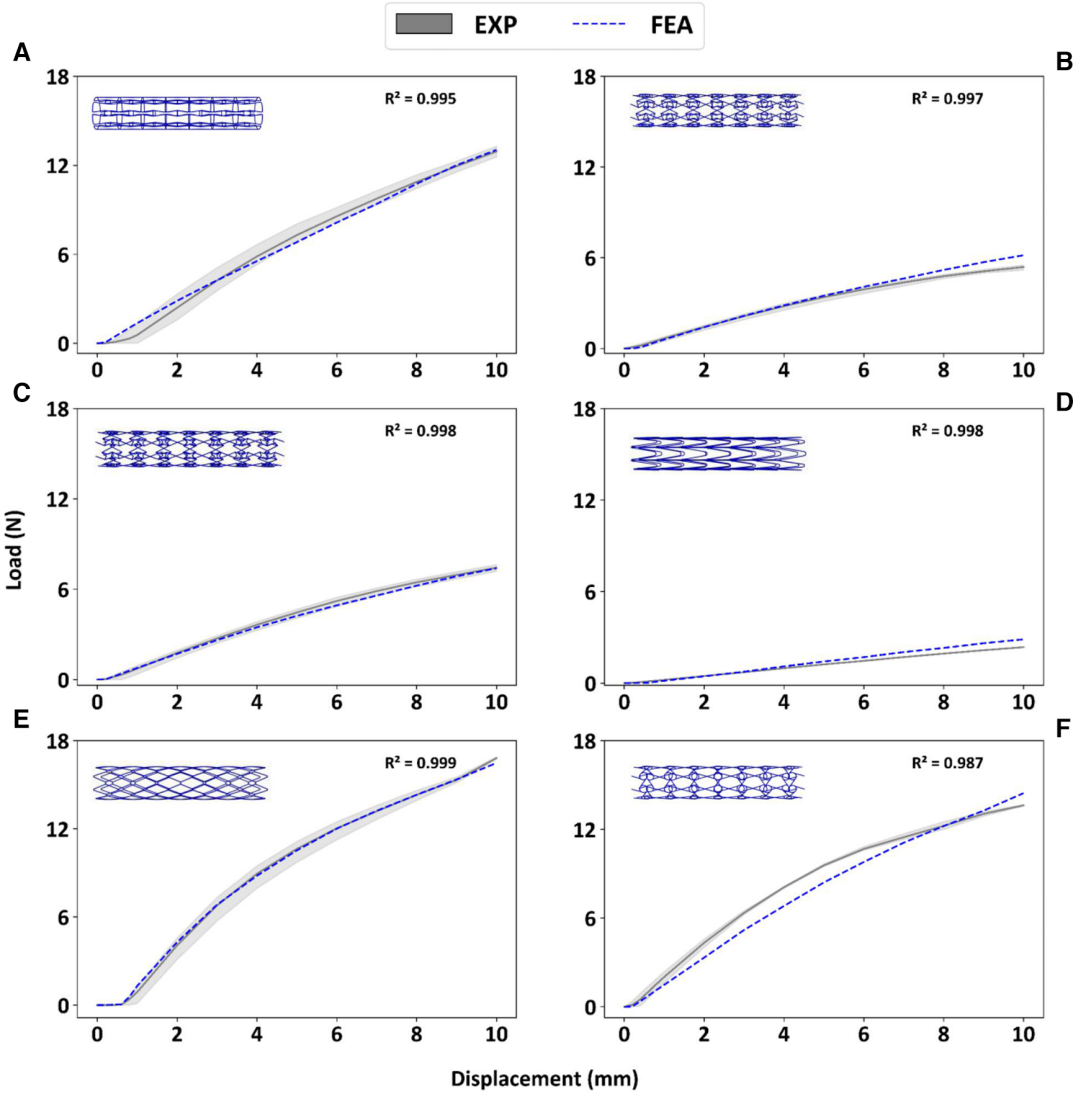
Experimental bending load vs. deflection curves are contrasted to corresponding FE results for prototypes RE (**A**), CRE (**B**), CS (**C**), HCB (**D**), DIA (**E**), CHIRAL (**F**).

**Figure 6:**
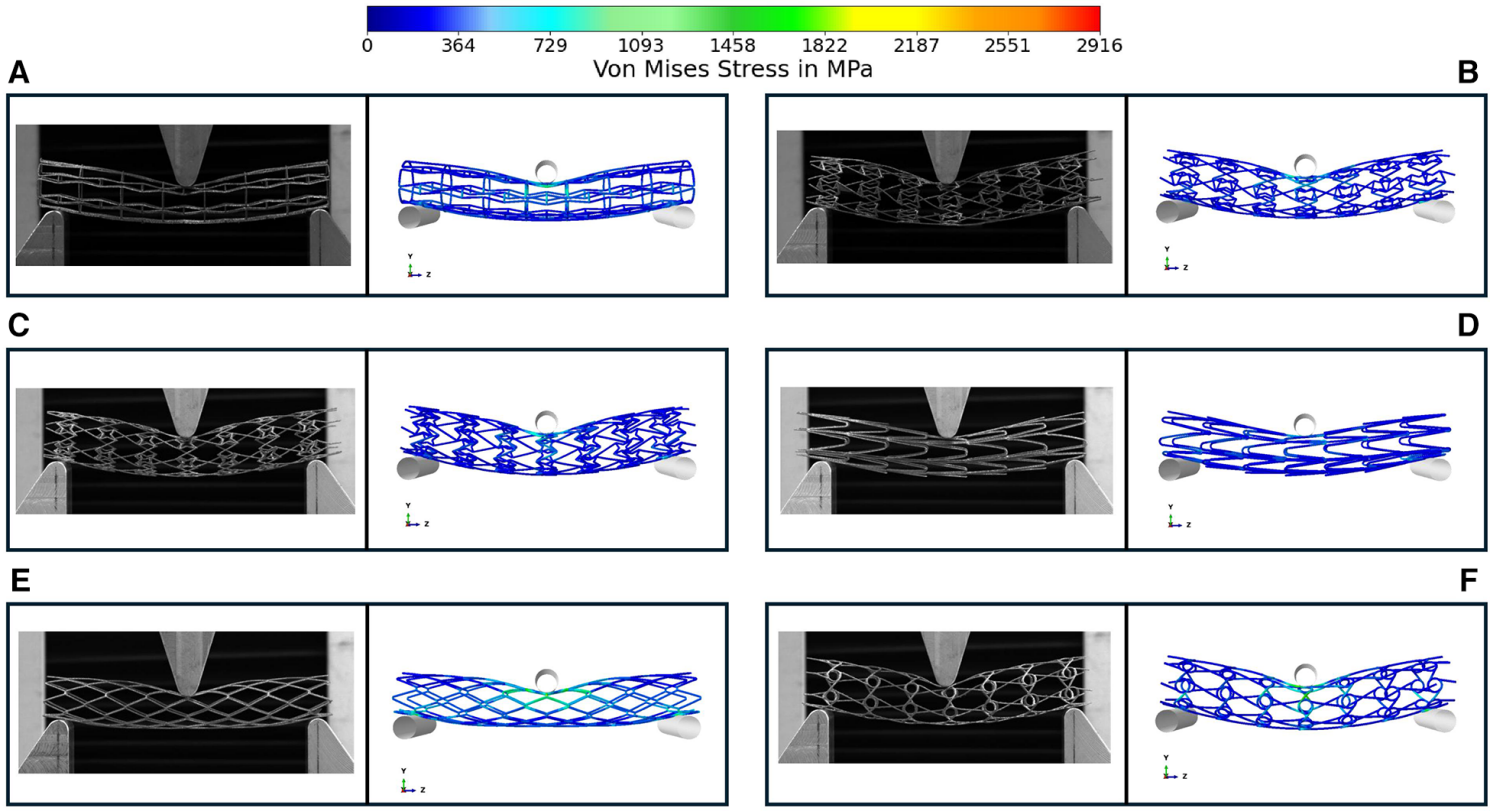
Comparison of bending deformation behavior in experimental testing and FE model for prototypes RE (**A**), CRE (**B**), CS (**C**), HCB (**D**), DIA (**E**), CHIRAL (**F**) the symmetrical FE model is mirrored in the Y & Z direction for comparison with three-point bend testing images. Images demonstrate the deformation at maximum deflection of 10 mm.

Figure 5 depicts the load-deflection curves of all stent designs extracted from experimental testing and FE models. Regarding the experimental tests, the average loading force was calculated from 3 prototypes of each stent design and the shaded area on all plots represents the standard deviation of the bending load magnitude for each stent design. Load-Deflection curves from the FE model closely correspond to those observed in the experimental tests for all stent designs with coefficient of determination (*R*^2^) magnitudes above 0.98 as seen in Table 4. Table 5 further reveals that the maximum error in predicting the mean experimental bending stiffness at a deflection of 10 mm using the FE model was 14% for the CRE stent design. Figure 6 demonstrates that the observed bending deformation characteristics visually align between the FE model and experiment. Thus, a good agreement between the FE model and experimental results can be corroborated by the qualitative features observed in Figure 6 and the quantitative criteria containing the coefficient of determination magnitudes and the overall maximum error rate mentioned previously.

**Table 4:** Coefficient of determination R^2^ magnitudes for FE model in predicting mean experimental results.

Stent design	*R* ^2^
RE	0.995
CRE	0.997
CS	0.998
HCB	0.998
DIA	0.999
CHIRAL	0.987

**Table 5:** Error estimation of FE model in predicting mean experimental bending stiffness at maximum deflection of 10 mm.

Stent design	Bending stiffness (*N*.mm^2^) at 10 mm deflection		Error percentage (%)
	Mean experimental	FE	
RE	10,488.99	10,578.23	0.84
CRE	4,372.44	5,005.19	14.47
CS	6,027.32	5,994.87	−0.54
HCB	1,914.46	2,165.94	13.14
DIA	13,644.62	12,687.39	−7.01
CHIRAL	11,056.84	11,722.04	6.02

There is a good agreement between the FE model and experimental results considering the similar bending deformation characteristics observed in Figure 6 and the previously mentioned quantitative criteria such as coefficient of determination magnitudes and maximum error rate overall. All stent prototypes exhibited maximum stress concentration at the indenter contact point during the bending test. The highest stress magnitude of 2,916 MPa was noted for Diamond stent design and lowest stress magnitude among all stent designs was observed to be 871 MPa for Honeycomb stent design.

## 4 Discussion

Some studies in the literature have extensively employed three-point-bending tests to assess the bending flexibility of auxetic tubular structures or auxetic stents, leveraging both FE simulations and experimental methodologies. Karnessis and Burriesci (27) utilized FE based numerical models to analyze the mechanical reaction of auxetic tubular structures (composed of inverted hexagonal honeycomb based unit cells) and demonstrated that these auxetic tubular structures under controlled design specifications can provide enhanced resistance to kinks under pure bending loading conditions. Lin et al. (12) conducted three-point bending tests on 3D printed NPR convex-concave honeycomb unit cell based stent and reported that their maximum load and displacement attributes post-test were higher than magnitudes for traditional stent designs of similar geometrical features and build material noted in literature. Liu et al. (28) applied three point bending tests to characterize the bending behavior of an indirect 3D printed novel porous stent comprised of a chiral auxetic frame for tracheal applications and demonstrated similar flexibility to native tracheal tissue.

Bhullar et al. (29) characterized the bending behavior of a femtosecond-laser cut manufactured stent with re-entrant hexagonal auxetic cell geometry using three-point bending test and reported that the auxetic stents displayed a dome morphology under bending loading conditions, thus enabling the stent to retain its patency until a significant magnitude of bending displacement. Bhullar et al. (29) also reconstructed the three-point-bending test with numerical models based on the FE method and noted a good agreement between experimental results and simulation results. Abbaslou et al. (17) examined the force-deflection curves of 3D printed RMCA stents using three-point-bending tests and noted a low error percentage between experimental and FE based numerical model results of 1.8%.

Although the bending behavior of individual stent designs derived from auxetic unit cells have been explored, there has been limited research work exploring the potential of DMLS AM process in printing metal based auxetic stents and validating numerical models for 6 different stent designs (4 Auxetic and 2 conventional) using experimental bending tests. To target this research gap, we modified the geometrical features of stent designs explored in Vellaparambil et al. (21) and utilized DMLS AM process in manufacturing prototypes for experiment testing using a three-point bend test protocol as per ASTM standard F2606-08 2021 (23).

While the primary purpose of the experimental testing in this work was to validate the FE model utilized in Vellaparambil et al. (21), the results of our three-point-bending tests reveal interesting insights regarding the suitability of DMLS process for producing auxetic metal stent prototypes. Considering that the original as-designed stents had a thickness of 0.4 mm which was at the lowest achievable thickness process specification for DMLS, we observed a deviation of ±5% in four stent designs as seen in Table 3, which was provided as input for the FE models of the mentioned stent designs. The presence of small support structure fragments were observed on CRE & CS stent prototypes as depicted in Figure 4, but no further post-processing actions were considered due to a high probability of stent fracture at those specific joints.

Inspired by the success of post-printing chemical etching technique for SLM-printed titanium stents in McGee et al. (26), we advocate for this cleaning method as a valuable solution to enhance surface quality, reduce strut oversizing, and overcome design limitations imposed by SLM/DMLS/LPBF processes for stent design in future research. Surface finishing techniques, such as etching, have the potential to improve the surface quality of stents by removing non-load-bearing surface defects. O'Keefe et al. (30) have shown that SLM fabricated etched Ti-6Al-4V lattice struts with 0.5 mm–0.7 mm strut thickness exhibit enhanced mechanical properties, including increased elastic modulus and tensile strength, compared to their non-etched counterparts due to the removal of non-load bearing surface defects. Finazzi et al. (31) observed a reduction in strut thickness of LPBF fabricated chemical etched nitinol stent specimens possessing 0.3 mm–0.6 mm strut thickness with no apparent changes in its super-elastic behavior and overall mechanical behavior post-etching. Based on above studies, by incorporating the calibrated material properties of the as-etched stents into a finite element (FE) model, we can anticipate a reduction in the error percentage when predicting the mean experimental bending stiffness of these etched stents post-processing. Additionally, the intricate joints of auxetic stent designs (CRE, CS, CHIRAL, RE) presented challenges regarding pre-printing support structure placement. AM rules outlined in Demir and Previtali (32) can be leveraged to optimize auxetic stent designs for 3D printing without introducing stiffness-enhancing features as observed in McGee et al. (24).

There is a good agreement concerning the load-deflection curves and bending deformation images extracted from the three-point-bending test for both experimental and FE model approaches as demonstrated in Figure 5 and Figure 6. The maximum error in predicting the mean experimental bending stiffness at the maximum bending deflection was 14%, could be influenced by the usage of Young's modulus (107 GPa) from literature (24) for the simplified titanium alloy material model. There is compelling evidence in recent studies (33, 34) involving Ti-6Al-4V based additively manufactured structures with thin struts demonstrating an approximate reduction of 50% in their mechanical properties in comparison to conventional mechanical properties of Ti-6Al-4V alloy mentioned in literature. Despite the limitation of not using a calibrated Young's modulus magnitude, the bending deformations from experimental results are captured in the FE model images as seen in Figure 6 with good accuracy. Figure 6 depicts all auxetic stent prototypes conformed to the shape of the loading indenter with the highest stress concentration occurring at the point of contact as noted in Bhullar et al. (29). The diamond stent design appears to transfer the stress distribution to surrounding regions from the point of contact in Figure 6, thus rationalizing its high bending load magnitude at the maximum bending deflection.

Simulation results in our previous work (21) based on a collection of different benchmark performance tests indicated that RE, CS, and CRE stent designs still stand out as promising candidates, consistent with their performance in experimental bending tests conducted in current work (Table 6). It is worth noting that the stent candidates in our initial study had a diameter of 30 mm, a strut thickness of 0.33 mm, and were constructed using Nitinol material. Force-displacement curves derived from the simulation results of three-point bend tests in our previous work (21) revealed that CHIRAL and DIA curves were closely positioned, mirroring the proximity of RE, CS, and CRE curves. Thus, we can infer that differences in Table 6 rankings between studies likely stem from variations in stent geometrical features and material properties.

**Table 6:** Stent designs are ranked 1 to 6 based on their bending flexibility, with 1 being the least flexible and 6 being the most flexible. This ranking is based on both simulated and experimental results from original study (21) and current work.

Ranking	Three-point-bend test (FE model: previous work)	Three-point-bend test (Experimental test: current work)	Three-point-bend test (FE model: current work)
1	CHIRAL	DIA	DIA
2	DIA	CHIRAL	CHIRAL
3	CRE	RE	RE
4	CS	CS	CS
5	RE	CRE	CRE
6	HCB	HCB	HCB

In conclusion, our finite element (FE) models effectively predicted the bending behavior of titanium alloy stent prototypes based on four different auxetic stent designs and two conventional stent designs, with experimental validation confirming their accuracy. The DMLS AM process demonstrates promising potential for fabricating auxetic stents with certain limitations concerning stent design, strut thickness and surface quality. However, further design related optimization using the AM guidelines established by Demir and Previtali (32) and post-processing involving chemical etching (25, 30, 31) to enhance surface quality and reduce strut thickness oversizing is recommended for future studies. AM processes hold the potential to create patient-specific stents incorporating auxetic unit cells, but their reliability and repeatability need to be enhanced through a controlled feedback loop based on future research that encompasses post-processing techniques and precise FE simulations to predict the behavior of printed stents. This approach can overcome current limitations in designing auxetic stents compatible with AM printing.

## Data Availability

The raw data supporting the conclusions of this article will be made available by the authors, without undue reservation.
